# A comparative study of the clinical efficacy and quality of life of modified Brisson surgery vs traditional Devine surgery in the treatment of concealed penis

**DOI:** 10.3389/fsurg.2025.1654258

**Published:** 2025-11-17

**Authors:** Na Guo, Bin Yang, Nan Li, Yi Wang, Haitao Chen

**Affiliations:** Department of Urology Surgery, Baoding Hospital, Beijing Children’s Hospital Affiliated to Capital Medical University, Baoding, Hebei, China

**Keywords:** modified Brisson surgery, concealed penis, clinical efficacy, traditional Devine surgery, quality of life

## Abstract

**Objective:**

This study aims to compare and analyze the differences in clinical efficacy and quality of life between the modified Brisson surgery and the traditional Devine surgery for the treatment of concealed penis (CP).

**Methods:**

This comparative study recruited 120 children with CP admitted to Baoding Hospital, Beijing Children's Hospital Affiliated to Capital Medical University, from March 2023 to July 2024. The patients were randomly divided into two groups: the experimental group and the control group, with 60 cases in each group. The experimental group received the modified Brisson surgery, while the control group received the traditional Devine surgery. The surgical outcomes, penile length before and after surgery, incidence of postoperative complications, and changes in quality of life were compared and analyzed between the two groups.

**Results:**

The response rate in the experimental group was 100%, which was significantly higher than that in the control group (93%), with a statistically significant difference (*P* = 0.02). At 6 months after surgery, the penile length in the experimental group was significantly longer than that in the control group (*P* < 0.001). The incidence of surgical complications was 5% in the experimental group and 17% in the control group, with a statistically significant difference (*P* = 0.03). Moreover, after intervention, the experimental group had significantly higher scores in indexes such as convenience of life, somatic feeling, negative emotion, self-concept, peer relationship, and self-satisfaction compared with the control group (*P* < 0.001).

**Conclusion:**

The modified Brisson surgery offers several advantages in the treatment of CP, including good treatment outcomes, a high response rate, a low postoperative complication rate, and significant improvements in quality of life indicators. These benefits make it a valuable approach worthy of promotion in clinical practice.

## Introduction

Concealed penis (CP) is a common pediatric genitourinary condition with an incidence of approximately 0.68%, ranking as the third most common congenital malformation of the urinary tract (1). In previous clinical practice, CP was often mistakenly treated with circumcision due to its misidentification as phimosis and redundant prepuce, leading to the failure of correct treatment for children (2). Internationally, there is no unified standard for the diagnosis of concealed penis, which hampers the preoperative surgical evaluation. With the progressive understanding of the etiology of this disease by physicians involved in the fields of urology, pediatric surgery, and plastic surgery, plastic surgery is generally used for the treatment of CP at the present stage. This situation of only a single surgical procedure has evolved into a situation where various surgical options are available due to many differences in scholars' understanding of the etiologic–pathologic structure of the disease, and correspondingly, the respective follow-up results vary greatly (3). To address the issue of skin coverage of the lengthened penis, CP is often treated clinically using surgical procedures such as the Devine, Brisson, and Johnston procedures. Postoperative prolonged tissue edema, particularly in the inner prepucial lamina, is a well-documented complication of CP surgery. For this reason, plastic coverage of the penile skin after penile degloving should be performed by utilizing the outer plate of the prepuce whenever possible. How to perform plastic coverage to achieve a satisfactory appearance is a major difficulty in CP surgery. Based on the principle of utilizing the outer plate of the prepuce as much as possible, a high priority has been placed on evaluating whether the outer plate skin is “sufficient,” which has led to the establishment of a penile skin plastic technique strategy for concealed penile orthopedics. Based on this technical platform, a specific individualized concealed penile orthopedic scheme is formulated by reconstructing the “orthogonal” penile angle. This study modified the Brisson technique by incorporating intraoperative digital measurements to optimize penile skin preservation, so as to avoid or reduce the occurrence of unfavorable factors. In addition, the clinical efficacy and quality of life of the modified Brisson surgery were compared with those of the traditional Devine surgery in the treatment of CP to evaluate the clinical efficacy of the modified Brisson technique and guide treatment decisions. The details are reported as follows.

## Materials and methods

### General data

A total of 120 children with concealed penis admitted to Baoding Hospital, Beijing Children's Hospital Affiliated to Capital Medical University from March 2023 to July 2024 were recruited and randomly divided into two groups: the experimental group and the control group, with 60 cases in each group. The experimental group received the modified Brisson technique, while the control group underwent traditional Devine surgery. The study was approved by the Institutional Ethics Committee of Baoding Hospital, Beijing Children's Hospital Affiliated to Capital Medical University (No.: 2023-05; 22 February 2023), and written informed consent was obtained from all participants.

Inclusion criteria: (1) children aged 3–16 years; (2) children who met the diagnostic criteria of concealed penis (4, 5); (3) children with normal testicular development and hormone levels; (4) children who volunteered to participate in the study and signed the consent form; (5) children who had no obvious mental disorder and were able to cooperate with the study. Exclusion criteria: (1) children with severe mental disorders who could not cooperate to complete the study; (2) children who cannot tolerate surgery because of other serious underlying diseases that cannot be corrected; (3) children with other penile deformities, such as webbed penis, micropenis, and trapped penis; (4) children with coagulation disorders.

In the experimental group, the age of the children ranged from 4 to 15 years, with an average of 6.73 ± 2.68 years, while in the control group, the age ranged from 6 to 15 years, with an average of 7.05 ± 1.81 years. No statistically significant differences were observed in baseline characteristics (*P* > 0.05; Table 1).

**Table 1:** Comparative analysis of general data of the two groups $\left(\bar{X}\pm S\right)$, *n* = 60.

Index	Experimental group	Control group	*t*	*P*
Age (years)	6.73 ± 2.68	7.05 ± 1.81	0.76	0.45
Duration of illness (years)	2.05 ± 0.22	2.02 ± 0.13	1.01	0.31
Body mass index (kg/m^2^)	21.07 ± 2.72	21.35 ± 2.41	0.60	0.55
Body weight (kg)	25.68 ± 2.70	25.27 ± 3.02	0.80	0.43
Resting length of penis (cm)	2.47 ± 0.70	2.38 ± 0.78	0.61	0.54
Stretched length of penis (cm)	5.83 ± 0.78	5.73 ± 0.82	0.68	0.50

*P* > 0.05.

### Methods

**Figure 1:**
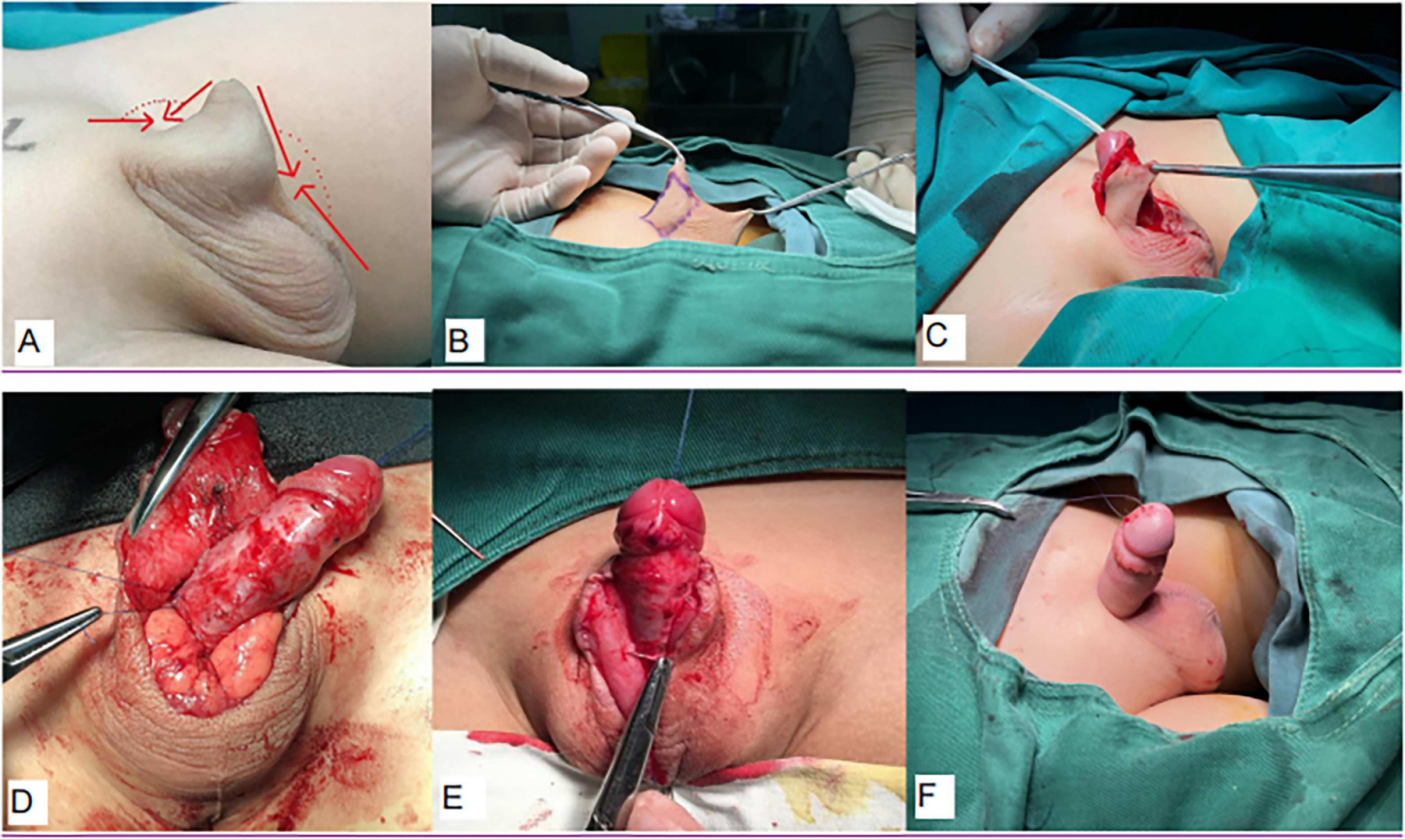
**(A)** Concealed penis: pyramidal appearance. **(B)** Surgical incision design. **(C)** Flap trimming. (**D**) Fixation of Buck's fascia to subcutaneous tissue. (**E**) Penis–scrotum angle reconstruction. (**F**) Post-suturing appearance.

Children in the experimental group underwent a modified Brisson surgery with a combination of digital measurements: first, the children were placed in the supine position, and an incision of approximately 3–5 mm was made in the middle of the dorsal side of the external prepuce mouth to fully expose the glans penis. The inner plate of the prepuce was circumcised 15 mm from the coronal groove. The prepuce was degloved along the Buck's fascia layer to the penile root. An inverted “V” incision was made at the junction of the ventral penis and scrotum to stretch the penis out from the incision. Subsequently, the cord-like fibers were removed from the dorsal side of the penis up to the superficial layer of the suspensory ligament, and the abnormal fascia tissue around the deep fascia of the penis was completely removed. The length of corpus cavernosum, the length of outer plate of the prepuce on the dorsal side of the penis, the length of outer plate of the prepuce on the ventral side of the penis, and the length of inner plate of the prepuce were measured. The designed suture was as follows: the length of outer prepuce plate on the dorsal side of the penis + the length of inner prepuce plate on the dorsal side of the penis = the length of corpus cavernosum. At the 3, 6, 9, and 12 o'clock positions of the penile root, Buck's fascia and subcutaneous tissue were fixed with 4-0 polydioxanone sutures (PDS-II) to establish 90° penopubic and penoscrotal angles, and the inverted “V” incision on the ventral side of penis was sutured in a “Y” or “Z” shape according to the specific situation. Finally, the frenulum of the prepuce was trimmed and formed, the redundant inner plate of the prepuce was removed, the inner and outer plates of the prepuce were circumferentially sutured, a double-lumen balloon catheter was indwelled, and the penis was bandaged with gauze under pressure (Figures 1A–F).

Children in the control group received the traditional Devine surgery: first, the children were anesthetized and placed in the same posture as the experimental group, and a 1 cm longitudinal incision was made on the dorsal side of the prepuce to separate the adhesion between the prepuce and the coronal groove of the glans penis until the narrow prepuce mouth was completely loosened. Subsequently, a lateral circumferential incision was made along the longitudinal incision on the dorsal side due to the traction of the penis. The superficial fascia of the penis was cut layer by layer, and the cord-like fibers around the penis were completely removed. The prepuce was degloved from the Buck’s fascia layer to the base, and the penis was fully pulled out and extended. Finally, the root of the penis was sutured and fixed with non-absorbable thread, the redundant prepuce was trimmed and then reset, and the prepuce was sutured intermittently at the inner and outer plates with absorbable sutures. The penis was indwelling for catheterization and bandaged with gauze under pressure. A catheter was indwelled, and the penis was bandaged with gauze under pressure.

### Observation indexes

(1) Comparative analysis of surgical outcomes: refer to the following standards according to the exposed length of the penis (6). Markedly effective: penile appearance resembling the appearance of circumcision, good reveal of the penis body, satisfactory penile scrotum angle and pubic penile angle, lengthened penile length of 2.5 cm or more, without recurrence. Effective: partial display of the penis body, satisfactory penile scrotum angle and pubic penile angle, lengthened penile length of l.5–2.5 cm with slight retraction. Improved: postoperative penile appearance from the front resembling the appearance of circumcision, complete retraction of the penis body viewed from the side, basic display of penile scrotum angle and pubic penile angle, penile coronal groove up to the ventral skin plane, lengthened penile length of <1.5 cm with retraction. Ineffective: penile appearance resembling the preoperative appearance from the lateral side, complete retraction and encapsulation of the penis body and the head of the penis under the skin, non-existence of the penile scrotum angle and the pubic penile angle, with complete recurrence. Overall response rate was calculated as: (markedly effective + effective cases) / total cases × 100%. (2) Comparison of penile length before and after surgery: the penile lengths of the two groups before and 6 months after surgery were compared, and the measurements were made by keeping a straightedge close to the pubic bone of the penis and measuring from the pubic bone to the glans of the penis. (3) Comparison of the incidence of postoperative complications between the two groups, such as infection, urethral injury, nerve injury, and preputial edema. (4) Quality of life: the Quality of Life Scale for Children and Adolescents (QLSCA) was used as the standard (7) to assess the quality of life of the two groups before and 6 months after surgery. The scale includes six dimensions: convenience of life, somatic feeling, negative emotion, self-concept, peer relationship, and self-satisfaction, with the higher score indicating a higher quality of life.

### Statistical analysis

All data in this study were statistically analyzed by SPSS 20.0 software, and measurement data were expressed as (±S). A two-independent sample *t*-test was used for comparison between the two groups, a paired *t*-test was used to analyze the data of the two groups before and after treatment, and the *χ*^2^ test was used for the comparison of rates. *P* < 0.05 indicates a statistically significant difference.

## Results

The comparative analysis of the surgical outcomes between the two groups showed that the overall response rate of the experimental group was 100%, which was significantly higher than 93% of the control group, with a statistically significant difference (*P* = 0.02) (see Table 2).

**Table 2:** Comparative analysis of surgical outcome indexes between the two groups $\left(\bar{X}\pm S\right)$, *n* = 60.

Group	Markedly effective	Effective	Improved	Ineffective	Overall response rate
Experimental group	43	17	0	0	60 (100%)
Control group	34	21	3	2	55 (93%)
*χ* ^2^					5.22
*P*					0.02

*P* < 0.05.

See Table 3 for the comparison of penile length before and after surgery between the two groups. No statistically significant differences were observed between the two groups in terms of penile length before treatment (*P* > 0.05). At 6 months after surgery, the penile length of the two groups was significantly improved compared with that before surgery (*P* < 0.001), and the improvement of the experimental group was more significant than that of the control group, with a statistically significant difference (*P* < 0.001).

**Table 3:** Comparative analysis of penile length before and after surgery between the two groups $\left(\bar{X}\pm S\right)$, *n* = 60.

Group	Before treatment	After treatment	*t*	*P*
Experimental group	2.47 ± 0.70	5.40 ± 0.62	24.36	<0.001
Control group	2.38 ± 0.49	4.40 ± 0.49	16.87	<0.001
*t*	0.61	9.81		
*P*	0.54	<0.001		

*P* < 0.05.

A comparison of the surgical complications between the two groups suggested that there were three cases (5%) of preputial edema without other complications in the experimental group, compared with seven cases (17%) of preputial edema and three cases (17%) of incision infection in the control group, with a statistically significant difference (*P* = 0.03). Neither group experienced any severe surgical complications such as urethral injury or nerve injury (Table 4).

**Table 4:** Comparative analysis of the incidence of surgical complications between the two groups $\left(\bar{X}\pm S\right)$, *n* = 60.

Index	Urethral injury	Incision infection	Nerve injury	Preputial edema	Total
Experimental group	0	0	0	3	3 (5%)
Control group	0	3	0	7	10 (17%)
*χ* ^2^					4.23
*P*					0.04

*P* < 0.05.

The scores of indexes such as convenience of life, somatic feeling, negative emotion, self-concept, peer relationship, and self-satisfaction in the experimental group after intervention were significantly higher than those in the control group, with statistically significant differences (*P* < 0.001) (Table 5).

**Table 5:** Comparative analysis of the quality of life scores between the two groups after intervention $\left(\bar{X}\pm S\right)$, *n* = 60.

Group	Convenience of life	Somatic feeling	Negative emotion	Self-concept	Peer relationship	Self-satisfaction
Experimental group	83.45 ± 5.10	85.17 ± 5.22	88.48 ± 5.56	85.82 ± 6.04	84.45 ± 5.30	76.93 ± 5.67
Control group	76.30 ± 5.35	73.65 ± 5.53	76.93 ± 4.83	78.30 ± 5.26	74.30 ± 4.61	71.27 ± 4.57
*t*	7.49	11.72	12.15	7.28	11.20	6.03
*P*	<0.001	<0.001	<0.001	<0.001	<0.001	<0.001

*P* < 0.05

## Discussion

Pediatric CP, a common clinical condition, occurs when the penis is inadequately exposed due to various factors, particularly congenital anatomical abnormalities such as fibrous band constriction during embryonic dysplasia, leading to a shortened distance between the preputial orifice and penile root (8). In addition, obesity and iatrogenic injury are also responsible for the occurrence of CP. Children with CP have normal development of the penile corpus cavernosum, with the penis body completely or incompletely concealed under the skin. This is mainly due to the gradual formation of cord-like tissue with poor elasticity during the development of the penis membrane, which leads to penis retraction (9). Affected children often present with glans abnormalities at the urethral meatus and prepuce early in life, which may impair penile development in adulthood. No unified etiology has been arrived at regarding concealed penis, and it is currently thought to be due to the inelastic myofascia that allows the fixation of the penile skin to the deep fascia, thus limiting the extension of the penis body (10). This is exacerbated by adhesions between the perineal and deep fasciae, along with localized thickening and reduced elasticity of elastic fibers, further worsening penile concealment (11).

One of the effective means of treating CP in children is surgery, whereby CP can be corrected for therapeutic purposes. Surgery for CP is recommended for most patients, preferably before preschool or adolescence. Given that CP can cause glans penis and, in severe cases, stenosis of the external orifice of the urethra, the absence of surgical treatment can cause psychological and physical disorders in affected children (12). There are currently several available surgical procedures for the correction of CP, including fixation of the penile skin, release of the fibrous cords of the penis body, and resection of prepubic fat in the lower abdomen. The purpose of these procedures is to remove the fibrous cords that affect the extension of the penis body, addressing the pathological changes of the concealed penis while restoring the penis to its normal shape (13). Although there are more available surgical options, they all have certain limitations (14). The Shirika surgery is performed purely to release phimosis caused by external orifice stenosis by cross-flap formation of the inner and outer plates. This procedure is not ideal for postoperative penile appearance and prevents the head of the penis from being naturally exposed. The Johnston surgery is to remove the stenosis by cutting the transverse seam longitudinally through the dorsal incision and fixing the penile skin with the root white membrane to correct the penile deformity. The Maizele surgery, after the stricture is relieved by dilation, is performed with a dorsal transverse incision to release and suture the foreskin and penis. Although these two surgical procedures solve the problem of penis exposure through fixation, they do not fundamentally solve the attachment and re-matching of the prepuce and penis. The Devine surgery and the Brisson surgery are common surgical approaches for the treatment of CP. The Devine surgery can improve the appearance of the penis in children to some extent, but it cannot meet clinical requirements due to obvious edema and the risk of damage to the vascular and nerve tracts (15).

The Brisson technique adheres to normal anatomical principles by excising abnormal penile root tissue to reduce redundant skin, ensuring proper fixation of penile skin to the shaft while minimizing tissue trauma. It contributes to the improvement of penis shape, prevention of penis retraction, amelioration of physiological function, and thus the quality of life of the child, ensuring normal blood supply to the body after surgery and reducing the occurrence of postoperative complications. In this study, the modified Brisson surgery was slightly modified under its original surgery method, and the foreskin incision was sutured first, and then a V-shaped incision was made, which was beneficial to the doctor's operation, thus avoiding the problem of repeatedly comparing the suture points when transferring the flap during the operation and effectively saving the operation time (16). At the same time, the improved Brisson surgery effectively fixed the anterior fascia and the white membrane of the pubic bone on both sides of the penis, which prevented the penis from shrinking after surgery and then made the penis grow effectively (17). The modified Brisson surgery can further narrow the root skin, make the buried penis appear further, and promote the penile skin to be better fixed on the penis body after removing the abnormal tissue on the dorsal side of the penile root, to solve the problem of foreskin shortage and improve the penile appearance (18). Our study demonstrated a 100% efficacy rate for the modified Brisson technique, while that of the traditional Devine surgery was 93% (*P* = 0.02). Six months after surgery, the penile length in the experimental group was significantly longer than that in the control group (*P* < 0.001). The incidence of surgical complications in the experimental group was 5%, while that in the control group was 17%, with a significant statistical difference (*P* = 0.03). It is suggested that the improved Brisson surgery is effective in treating CP. During surgery, the length of corpus cavernosum, the length of outer plate of the prepuce on the dorsal side of the penis, the length of outer plate of the prepuce on the ventral side of the penis, and the length of inner plate of the prepuce were measured. The redundant inner prepuce plate was removed, and the inner and outer plates of the prepuce were annularly sutured. Due to scientific design and reasonable length of inner and outer plates of the prepuce, the incidence of complications such as preputial edema and incision infection was obviously reduced. However, no serious surgical complications such as urethral injury and nerve injury occurred in both groups.

The modified Brisson surgery with a combination of digital measurements can effectively prevent intractable preputial edema. It can improve the beauty of the penis and promote the growth of the penis (19). A study by Sol Melgar et al. (20) revealed that the penile growth length of children with concealed penis treated by the improved Brisson surgery was longer with a more satisfactory appearance. Moreover, the recovery time of postoperative edema and bandage dressing time were shorter than those in the conventional group. Our study confirmed significantly higher postoperative scores in the experimental group for convenience, somatic comfort, emotional well-being, self-concept, peer relationships, and satisfaction (*P* < 0.01) It is suggested that the improved Brisson surgery can effectively reduce the degree of preputial edema in children with concealed penis, promote penile growth, and improve the quality of life indicators such as somatic feeling, negative emotion and self-satisfaction.

To put it in a nutshell, the improved Brisson surgery boasts various advantages in the treatment of concealed penis, such as improved quality of life, promoted penile growth, reduced degree of preputial edema, reduced incidence of postoperative complications, and increased satisfaction. It is effective in the treatment of an occult penis and can provide an effective reference for clinical treatment of a concealed penis. Nevertheless, certain shortcomings are still visible in this study: this study is a single-center study with a small sample size and a short follow-up time. In view of this, the sample size will be further increased, multicenter research will be carried out, and the follow-up time will be extended in future clinical studies, with a view to evaluating the advantages and disadvantages of this treatment scheme more objectively.

## Data Availability

The raw data supporting the conclusions of this article will be made available by the authors, without undue reservation.
